# Different Growth Responses of an Invasive Weed and a Native Crop to Nitrogen Pulse and Competition

**DOI:** 10.1371/journal.pone.0156285

**Published:** 2016-06-09

**Authors:** Ping Lu, Jingxin Li, Chenggong Jin, Baiwen Jiang, Yamei Bai

**Affiliations:** 1 College of Resources and Environmental Sciences, Northeast Agricultural University, Harbin, China; 2 College of Animal Science and Technology, Northeast Agricultural University, Harbin, China; University of A Coruña, SPAIN

## Abstract

Resource pulses are a common event in agro-ecosystems. A pot experiment was conducted to assess the effects of nitrogen (N) pulses and competition on the growth of an invasive weed, *Amaranthus retroflexus*, and a native crop, *Glycine max*. *A*. *retroflexus* and *G*. *max* were planted in pure culture with two individuals of one species in each pot and in mixed culture with one *A*. *retroflexus* and one *G*. *max* individual and subjected to three N pulse treatments. The N treatments included a no-peak treatment (NP) with N applied stably across the growing period, a single-peak treatment (SP) with only one N addition on the planting date, and a double-peak treatment (DP) with two N additions, one on the planting date and the other on the flowering date. N pulse significantly impacted biomass and height of the two species across the whole growing season. However, only the relative growth rate (RGR) of *A*. *retroflexus* was significantly affected by N pulse. *A*. *retroflexus* had the greatest biomass and height in the SP treatment at the first harvest, and in the DP treatment at the last three harvests. Pure culture *G*. *max* produced the greatest biomass in the DP treatment. In mixed culture, *G*. *max* produced the greatest biomass in the NP treatment. Biomass production of both species was significantly influenced by species combination, with higher biomass in mixed culture than in pure culture at most growth stages. Relative yield total (RYT) values were all greater than 1.0 at the last three harvests across the three N treatments, suggesting partial resource complementarity occurred when *A*. *retroflexus* is grown with *G*. *max*. These results indicate that *A*. *retroflexus* has a strong adaptive capacity to reduce interspecific competition, likely leading to its invasion of *G*. *max* cropland in China.

## Introduction

Invasion by exotic species is one of the most significant threats to biodiversity and ecosystems globally [1]. Understanding the mechanisms by which invasive species outcompete native species is necessary to reduce the negative impacts of the invasive species. Dozens of hypotheses have been put forward to explain the success of invasive species [2]. One hypothesis to explain plant invasions is the Fluctuating (pulses) Resource Availability Hypothesis (FRAH). The FARH posits that invasion is promoted by high resource availability, which in turn due to disturbance or low resource absorption by the native species [3].

Recent studies have tested the FRAH, and investigated the relationship between nitrogen (N) pulses and invasive species success [4–8]. Work by Huenneke *et al*. [4], Vasquez *et al*. [5] and Esque *et al*. [6] found that invasions by exotic species in California serpentine grasslands, western United States grasslands, and Mojave Desert shrublands, respectively, were accelerated by N addition. In opposition to the FRAH, Harrison [7] found that invasive species richness had no relationship to soil N levels in a grazed California grassland, while Funk & Peter [8] found in resource-limited environments in Hawaii Volcanoes National Park, invasive plants often exceeded native species in resource-use efficiency (RUE) for a brief period, whereas RUE was similar between the invasive and native species over leaf lifespan period. Together, the results of these studies suggest that the relationship between N pulse and invasive plant performance depends on habitat and the invasive plant species.

Agro-ecosystems are a common habitat of invasive species, and more than half of the invasive species in China are found in agro-ecosystems [9]. Resource pulses are also common in agro-ecosystem, and are more frequent and greater in agro-ecosystem than natural ecosystems due to watering, weeding, fertilizer application, and shifts of crop species. Invasive weed species face strong and fluctuating selection pressures that arise from changes in farmland resulting in high adaptability [10]. Although research exists on the mechanisms of invasive weeds [11–12], studies on how invasive weeds adapt to resource pulses in agro-ecosystems are lacking, and few studies consider the competition between invasive weeds and native crops.

Nitrogen is one of the most important nutrients applied to improve crop yield [13]. The timing of application and amount of N fertilizer can impact competition between weed and crop species [14–16]. For example, competition between *Stellaria media* and wheat increased with higher N levels, but the opposite result was observed with potato crops [14]. Additional research found that the competitive ability of *Lolium rigidum* was lower when N was applied early in the growing season than later stages [15]. *Veronica hederifolia* was less competitive when N was applied at the stem elongation stage of winter wheat compared to the tilling stage [16]. The competitive ability of weeds often changes with soil N availability and may vary with the weed species and crop. However, few studies have investigated the effects of variable N pulses due to variable N fertilization regimes on the competition between invasive weeds and crop species.

This study was carried out to assess the influence of N pulses on the growth of an invasive weed and a native crop. *Amaranthus retroflexus* is native to America and is one of the most invasive weeds in China [17], *Glycine max* is an important crop in China is native to north and central China [18]. *A*. *retroflexus* is often found in *G*. *max* fields throughout China [19]. In the following study, *A*. *retroflexus* and *G*. *max*, which co-occur in farmland in northern China were planted in either pure culture or mixed culture to assess the influence of variable N pulses on the invasive capacity of *A*. *retroflexus*. We hypothesize that (1) in pure culture, *A*. *retroflexus* has a greater growth response to N pulses (the SP and DP treatments) than *G*. *max*; (2) in mixed culture, the competitive ability of *A*. *retroflexus* is higher in SP and DP treatments than in NP.

## Materials and Methods

### Study species

Indigenous to America, *A*. *retroflexus*, a C_4_ annual weed, is one of the most widely spread invasive species in China [20], and occurs in temperate and subtropical areas of the world [21]. It has been in China for more than 150 years with the first recorded observation in the middle of 19th century [20]. It is commonly found in wastelands and disturbed areas including roadsides, riverbanks, gardens, farmlands, and fallow lands. It germinates in late spring and bears seeds in late summer or early autumn. It has an upright stem, with a height of between 0.05 m and 2 m, and generally branched but can also be simple depending on plant density. In the Northern Hemisphere, it normally blossoms and bears seeds from July to September [22]. A branched indeterminate inflorescence can bear many thousands of flowers producing 10 000–300 000 small seeds. Seeds are wind dispersed. Removal of this species is difficult due to the number of seeds in the seedbank [23].

Indigenous to China, *G*. *max* is a C_3_ crop species and is one of the most significant crops in China [24]. It is a green, bushy legume and is often invaded by *A*. *retroflexus* in China [19]. It is commonly planted in spring and harvested in autumn [24]. The *G*. *max* cultivar used in this study is Northeast Agricultural University 54, with indeterminate growth habit, 0.8–1.0 m high [25]. The three northeast provinces (Heilongjiang, Jilin, and Liaoning) are the greatest producing yield regions of *G*. *max* in the country. Heilongjiang is the greatest *G*. *max* producing area.

### Experimental design

This study was carried out at the Northeast Agriculture University in Harbin, China (45°34′N, 126°22′E). Mean annual precipitation is 590 mm, with peak precipitation in July and August. Mean annual temperature is 4.5°C, and mean monthly air temperatures ranging from -17.1°C in January to 23.4°C in July. The Heilongjiang Meteorological Administration of China provided the meteorological data.

Seeds of the two species were gathered in the Xiangfang Farm Experiment Base of the Northeast Agriculture University. The soil was a typical black loam soil with a pH of 7.57 and 2.31 mg g^-1^ of organic matter. Total N content was 0.015 mg g^-1^. Soil was passed through a 5 mm sieve and mixed thoroughly, placed in 30 cm diameter by 30 cm height plastic pots. Each pot contained 12.75 kg dry soil, and surface watered to bring the soil water content to field capacity. Four to six seeds of each species were planted on May 17, 2011 and were subsequently thinned to two plants (pure culture: two individuals of one species, or a mixed culture: two individuals containing two different species) per pot within 1 week of emergence.

A factorial design was carried out with six replicates in randomized blocks. The two factors were: N pulse and species composition. The experiment consisted of three N treatments: the no-peak treatment (NP) with N applied stably across the growing period, the single-peak treatment (SP) with only one N addition on the planting date (May 17, 2011), and the double-peak treatment (DP) with two N additions, one on the planting date, the other on the flowering date (July 2, 2011). The experiment contained nine treatments (three N pulses × three species compositions). Each block consisted of six replicate sample pots per treatment for monthly destructive sampling. There were six blocks altogether. All of the three N treatments were the same total quantity at 50 kg·ha^-1^, which is representative of N fertilization levels in *G*. *max* fields in northeast China. Nitrogen was hand incorporated as urea (CO(NH_2_)_2_) into pots based on the surface area of the pot. In the SP treatment, the plants were supplied with 0.5 L urea (CO(NH_2_)_2_) at a concentration of 26.22 mM once on the planting date. In the DP treatment, the plants were supplied with 0.5 L urea (CO(NH_2_)_2_) at a concentration of 13.1 mM twice, once on the planting date, the once on the flowering date. In the NP treatment, the plants were supplied with 0.5 L urea (CO(NH_2_)_2_) at a concentration of 2.016 mM every 10 days over the duration of the experiment. Phosphorus and potassium were applied at a rate of 50 kg·ha^-1^, respectively, all at once on the planting date. To ensure water was not a growth-limiting factor, plants were watered at least once daily.

### Measurement of biomass and height

The six pots in each of the nine treatments were chosen for biomass measurement by harvesting every 30 days during the growing period. At harvest, the height of *A*. *retroflexus* and *G*. *max* was recorded and shoot parts were divided from root parts by cutting the shoots at the soil surface. The soil was carefully removed from the root system. The roots were thoroughly rinsed. Shoots and roots were weighed after oven drying at 65°C for at least 48h. The relative growth rate (RGR) was evaluated as percent variation of plant biomass over a 30-day period.

### Competition analysis

#### Relative yield per plant (RYP)

The relative yield per plant (RYP) of each species was used to evaluate the species competitive effects, which were calculated as:

RYPij = YPij/YPii

YPij is the biomass production of species i in mixed culture with species j and YPii is the biomass production of species i in pure culture. RYPij > 1 indicates that the individual species i reacts positively to competition with the individual species j. RYPij < 1 indicates that species i reacts negatively to competition with species j [26]. Species with high competitive capacity have high values of RYP [27].

#### Relative yield total (RYT)

The relative yield total (RYT) is an assessment of the interaction type of species mixture, which was calculated as:

$${\text{RYT }=\text{ RY}}_{\text{ij}}+{\text{RY}}_{\text{ji}}\;=\;\left(\text{Yij/Yii}\right)\text{+}\left(\text{Yji/Yjj}\right)$$

Yii (or Yjj) is the yield of species i (or j) when growing in pure culture per pot, Yij (or Yji) is the yield of species i (or j) when growing with species j (or i) per pot.

RYT = 1 suggests that the species are competing for the same limiting resources. RYT > 1 suggests that the species have different resources requirements or are benefiting from the interaction. RYT < 1 suggests a negative interaction aside from competition such as allelopathy [28].

### Data analysis

Statistical analyses were carried out using a three-way repeated measures analysis of variance (RMANOVA) (Procedure in SPSS 17.0, USA) to test the main effects and interactions of N treatment, competition and sampling dates on biomass, height, and RGR in *A*. *retroflexus* and *G*. *max* during the growing seasons. A two-way repeated measures analysis of variance (RMANOVA) was run for each species to determine the effects of N treatment, sampling dates and the N treatment × sampling dates on relative yields and relative yield total. A Duncan’s multiple range test was used to examine the differences between treatments. One-sample t test was used to compare the differences in RY_GA_, RY_AG_, and RYT of each treatment from 1. An independent sample t test was used to test the differences in total biomass, height and RGR between the two species in each treatment, and the differences between RY_GA_ and RY_AG_ in each treatment. All data met the homogeneity of variance so data transformation was not required.

## Results

### Biomass

**Fig 1:**
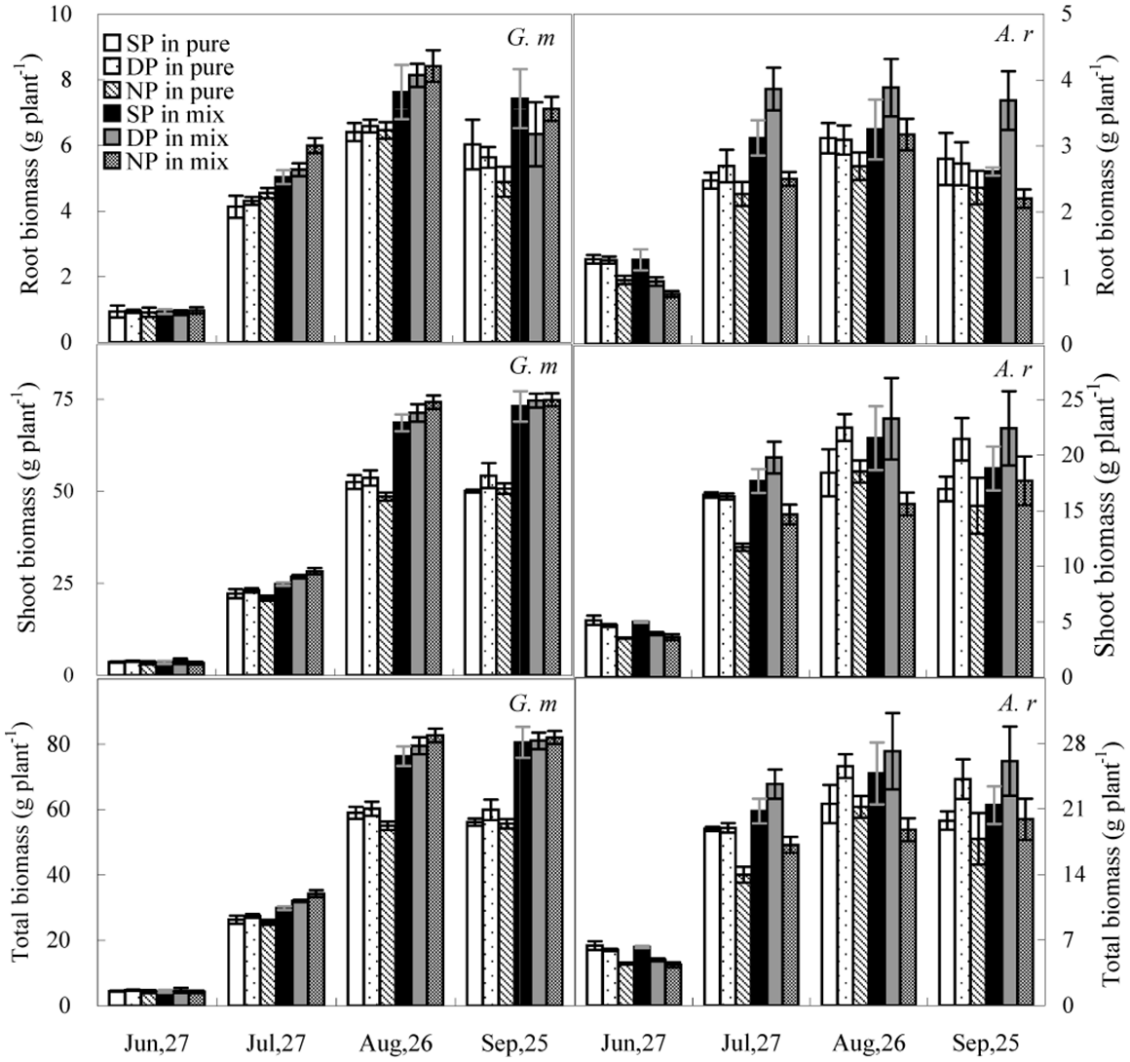
Changes in root, shoot, and total biomass of *A*. *retroflexus* (*A*. *r*) and *G*. *max* (*G*. *m*). Plants were grown in pure culture (pure) or mixed culture (mix) in the three N pulsed treatments (SP, single-peak treatment; DP, double-peak treatment; NP, no-peak treatment). All values are the average ±S.E.

Biomass progressively increased for both species during the first three growth stages and then decreased in the final growing stage (Fig 1). By the first harvest, total biomass of *A*. *retroflexus* was greater than *G*. *max* in the SP and DP treatments (*P* < 0.001), but at the last three harvests total biomass of *G*. *max* exceeded *A*. *retroflexus* (*P* < 0.001).

RMANOVAs on shoot, root, and total biomass of both species at each harvest showed a significant N pulse effect at all growth stages with the exception of *G*. *max* root biomass (*P* < 0.05; Table 1). *A*. *retroflexus* individuals grown in the SP treatment had higher (*P* < 0.05) shoot, root, and total biomass compared to individuals grown in the DP and NP treatments at the first growth stage. However, in the last three growth stages *A*. *retroflexus* individuals grown in the DP treatment had higher (*P* < 0.05) total biomass compared SP and NP treatments. Throughout the whole growing period, shoot, root, and total biomass were greater for *G*. *max* individuals grown in the DP treatment than in the SP and NP treatments in pure culture (*P* < 0.05; Fig 1). Pure culture *G*. *max* had 7% and 8% higher (*P* < 0.05) final total biomass in the DP treatment than in the SP and NP treatments, respectively. Differences in shoot, root, and total biomass of *G*. *max* in mixed culture were not detected among the three N treatments at the first harvest, while *G*. *max* total biomass in mixed culture were 2–14% and 1–7% higher in the NP treatment compared to the SP and DP treatments, respectively, at the last three harvests (*P* < 0.05).

**Table 1:** F-values for the effects of nitrogen pulses (N), competition (C), and sampling date (D) on root, shoot, total biomass (Total), height (H), and relative growth rate (RGR) in *A*. *retroflexus* and *G*. *max* (Three-way RMANOVA).

	Root	Shoot	Total	H	RGR
*A*. *retroflexus*					
**C**	40.507***	25.042***	32.503 ***	8.400**	5.061^ns^
**N**	68.731***	125.794***	135.631***	40.847***	17.302***
**D**	555.925***	604.583***	657.122***	4630.656***	5667.09***
**C×N**	15.284***	1.547^ns^	2.136^ns^	2.601^ns^	0.097^ns^
**D×C**	21.746**	4.858**	6.208**	0.975^ns^	39.647***
**D×N**	7.22***	7.489***	7.671***	21.065***	24.136***
**C×N×D**	17.016 ***	3.394**	2.602 *	0.738^ns^	12.812***
*G*. *max*					
**C**	232.022***	1558.615***	1496.457***	3.911^ns^	67.177***
**N**	1.643^ns^	10.049***	7.666**	5.253**	0.271^ns^
**D**	1487.49***	1498.349***	1466.824***	6749.412***	2670.25***
**C×N**	8.549**	18.67***	19.966***	0.514^ns^	0.341^ns^
**D×C**	25.549***	430.93***	393.344***	2.472^ns^	29.437***
**D×N**	7.121***	1.52^ns^	1.364^ns^	2.609*	7.587**
**C×N×D**	1.840^ns^	6.294***	5.928***	0.925^ns^	3.504**

^ns^, Not significant (*P >* 0.05);

*P < 0.05;

**P < 0.01;

***P < 0.001.

Root, shoot, and total biomass of both species were significantly affected by competition and date, and a significant interaction between competition and date was found (*P* < 0.01; Table 1). Differences between pure culture and mixed culture were not detected at the first harvest for root, shoot, and total biomass of the two species (except for *A*. *retroflexus* individuals grown in the DP treatment). However, at the three subsequent harvests the three biomass parameters (with the exception of *A*. *retroflexus* in the NP treatment at the third harvest) were significantly higher in mixed culture than in pure culture (*P* < 0.05). At the time of the second harvest, root, shoot, and total biomass was 9–43%, 8–25%, and 10–25% greater, respectively, for *A*. *retroflexus* individuals grown in mixed culture than pure culture in all three N treatments (*P* < 0.001). At the same time, root, shoot, and total biomass was 22–32%, 12–35%, and 13–35% greater, respectively, for *G*. *max* plants grown in mixed culture (*P* < 0.05), than pure culture in all three N treatments (Fig 1).

### Height and relative growth rate (RGR)

**Fig 2:**
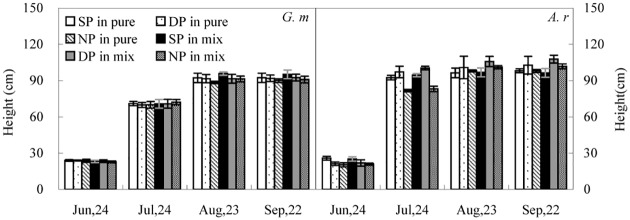
Changes in height of *A*. *retroflexus* (*A*. *r*) and *G*. *max* (*G*. *m*). See Fig 1 for abbreviations. All values are the average ±S.E.

At the first harvest, plant height was greater in *A*. *retroflexus* than in *G*. *max* in the SP treatment (*P* < 0.05), but was slightly lower in the DP and NP treatments (*P* <0.05). During the remainder of the growing season, plant height was greater in *A*. *retroflexus* (*P* < 0.05) (Fig 2).

N pulse significantly affected plant height of *A*. *retroflexus* and *G*. *max* at all growth stages (*P* < 0.01; Table 1). By the first harvest, *A*. *retroflexus* height was 16%-21% and 21–27% (*P* < 0.05) higher in the SP treatment compared to the DP and NP treatments, respectively. In the last three stages *A*. *retroflexus* height was 5%-11% and 3–21% (*P* < 0.05) higher in the DP treatment compared to the SP and NP treatments, respectively. *G*. *max* height did not vary among the three N treatments at the first and second harvests, but in the SP treatment at the last two harvests plant height was 0.5%-5% and 3–5% (*P* < 0.05) higher than that in the DP and NP treatments, respectively. Height of *A*. *retroflexus* was significantly affected by competition at all stages of growth (*P* > 0.01), but competition did not affect the height of *G*. *max* (*P* > 0.05; Table 1).

**Fig 3:**
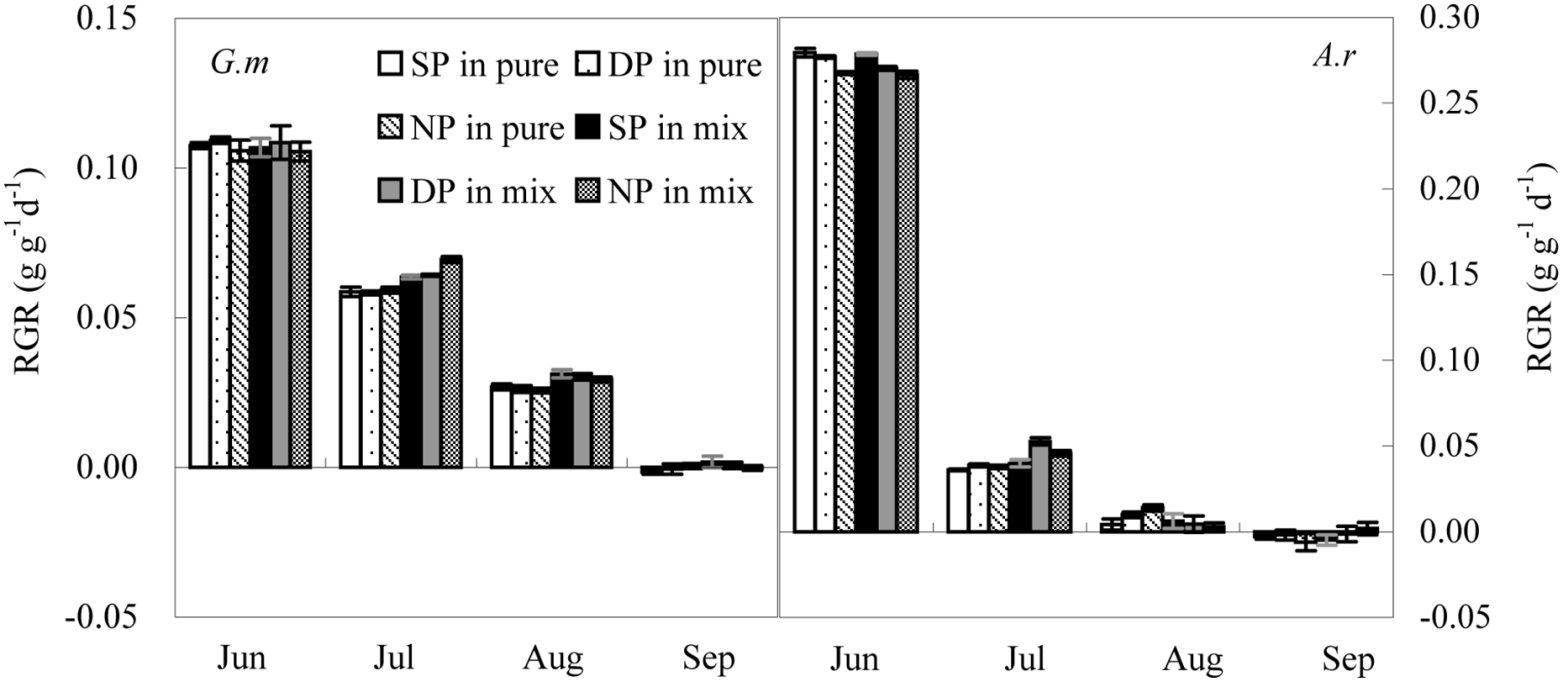
Changes in Relative growth rate (RGR) of *A*. *retroflexus* (*A*. *r*) and *G*. *max* (*G*. *m*). See Fig 1 for abbreviations. All values are the average ±S.E.

The same seasonal pattern was observed in relative growth rate (RGR) for *A*. *retroflexus* and *G*. *max* in all N treatments and competition treatments. The greatest RGR was recorded during the first growth stage and then decreased progressively across the last three growth stages (Fig 3). Initial RGR was significantly greater in *A*. *retroflexus* than in *G*. *max* (*P* < 0.001), but thereafter RGR of *A*. *retroflexus* was lower than that of *G*. *max* (*P* < 0.05). RGR of *A*. *Retroflexus* declined more rapidly than *G*. *max* after the first harvest likely due to its early reproduction.

RGR of *A*. *retroflexus* was significantly impacted by N pulse (P < 0.001), while *G*. *max* was not (*P* > 0.05; Table 1). At the first harvest, *A*. *retroflexus* individuals grown in the SP treatment had 1%-3% and 4–5% higher RGR (*P* < 0.05) than individuals grown in the DP and NP treatments, respectively. At the second harvest, *A*. *retroflexus* individuals grown in the DP treatment had 7%-32% and 2–15% higher RGR (*P* < 0.05) than individuals grown in the SP and NP treatments, respectively. Interactions between competition and sampling date significantly affected RGR of both species (*P* < 0.001; Table 1). *A*. *retroflexus* and *G*. *max* individuals grown in mixed culture had slightly lower RGR than individuals grown in pure culture at the first harvest but individuals grown in mixed culture had 8–17% and 11–35% higher RGR (all *P* < 0.001), respectively, than individuals grown in the pure culture at the second harvest.

### Relative yield per plant (RYP) and relative yield total (RYT)

**Fig 4:**
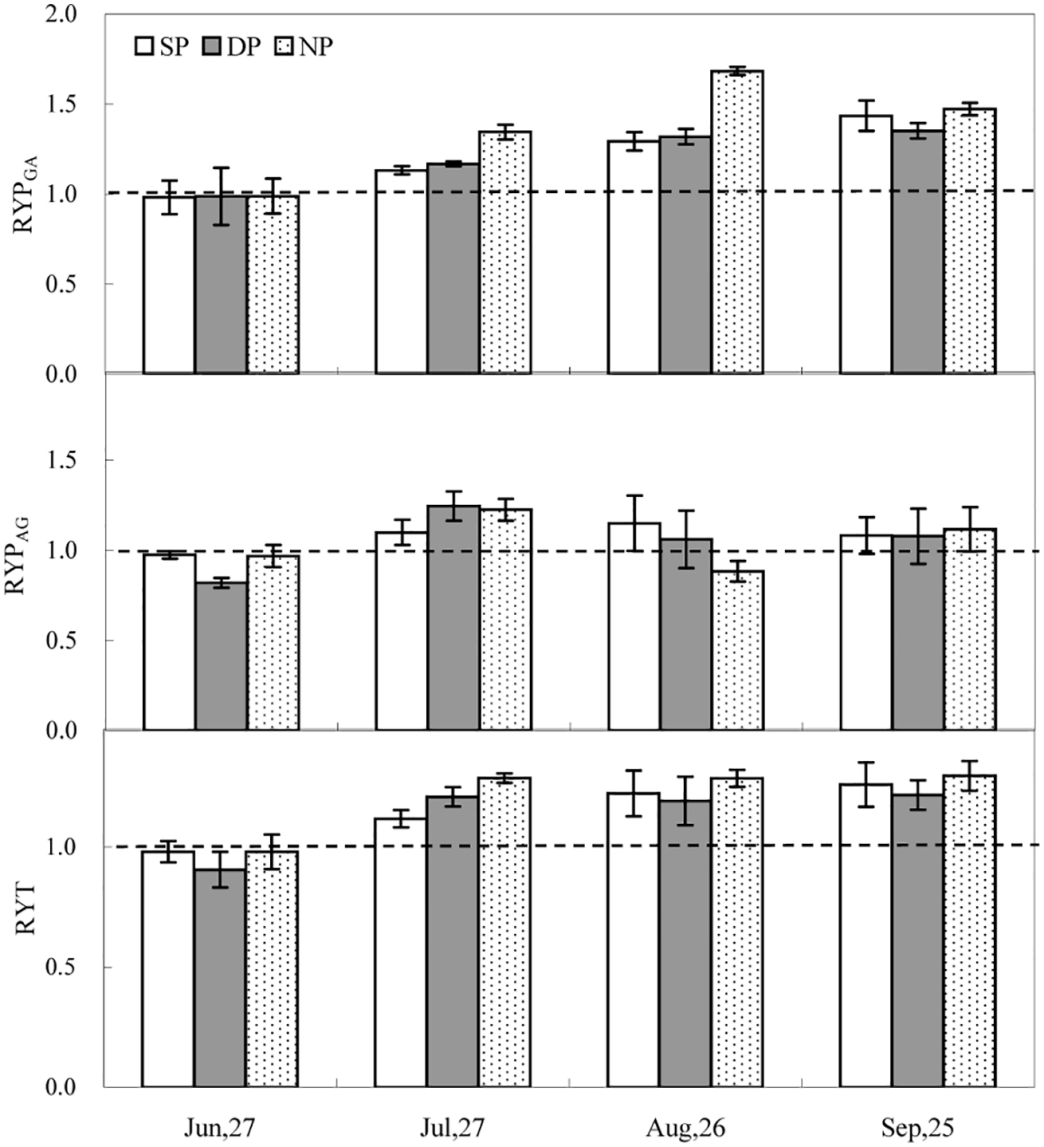
Relative yield per plant (RYP) and relative yield total (RYT) of *A*. *retroflexus* (*A*. *r*) and *G*. *max* (*G*. *m*). RYP_GA_: relative yield per plant of *G*. *max* with *A*. *retroflexus*. RYP_AG_: relative yield per plant *A*. *retroflexus* with *G*. *max*. See Fig 1 for abbreviations. All values are the average ±S.E.

RYP_GA_ was significantly affected by N pulse (*P* < 0.001; Table 2). RYP_GA_ did not vary among the three N treatments at the first harvest (*P >* 0.05), while RYP_GA_ was 13–34% and 12–33% greater in the NP treatment compared to the SP and DP treatments, respectively, at the second and third harvests (*P* < 0.05). RYP_GA_ in the SP and NP treatments was greater than the DP treatment at the last harvest (*P* < 0.05). N pulse and sampling date interacted to affect RYP_AG_ (*P* < 0.001) (Table 2, Fig 4). At the first harvest, RYP_AG_ in the SP and NP treatments was greater than in the DP treatment (*P* < 0.05). At the second harvest, RYP_AG_ in the DP and NP treatments was greater than that in the SP treatment (*P* < 0.05). At the third harvest, RYP_AG_ in the SP and DP treatments was greater than that in the NP treatment (*P* < 0.05). However, RYP_AG_ did not differ among the three N treatments at the final harvest (*P >* 0.05).

**Table 2:** F-values for the effects of nitrogen pulses (N) and sampling date (D) on relative yield per plant (RYP) and relative yield total (RYT) in *A*. *retroflexus* (A) and *G*. *max* (G) (Two-way RMANOVA).

	RYP_GA_	RYP_AG_	RYT
**N**	30.099***	0.908^ns^	10.087**
**D**	181.22***	20.087***	81.504***
**D×N**	13.113***	5.597***	2.475*

^ns^, Not significant (*P >* 0.05);

*P < 0.05;

**P < 0.01;

***P < 0.001.

RYT values for total biomass production ranged from 0.91 to 1.28. RYT values were all higher than 1.0 in the last three growth stages (*P* < 0.05) (Fig 4). RYT values greater than 1.0 suggests that the yield is improved when *A*. *Retroflexus* and *G*. *max* are grown in mixed culture. RYT values were significantly affected by N pulse, sampling date, and a significant interaction between the two factors was detected (Table 2).

## Discussion

### Responses to N pulses

Disturbance is an important ecological phenomenon that can be used to explain successful invasion of non-native species [29] as well as resource fluctuations [3]. The FARH suggests that temporary increases in resource availability, such as water and nutrients, caused by disturbances [3] provide chances for invasive plants to establish in native ecosystems. However, previous studies on how invasive plant biomass production responds to N pulses have obtained differing results. Todd *et al*. [30] found that N pulses were more beneficial to biomass production of invasive annual plants than that of native annual plants. While James *et al*. [31] reported that the invasive grass *Schismus arabicus* accumulated more biomass with consecutive N additions rather than pulses of N additions. Finally, Olson & Blicker [32] documented that under pulses of N addition, the invasive plant *Centaurea maculosa* took up less N than one native plant *Pascopyrum smithii* but more than another native plant *Pseudoroegneria spicata*, and produced more biomass than *P*. *spicata*, but less than *P*. *smithii*. These results suggest that, the effect of N pulses on invasive plant biomass depends on the occurrence time and size of the N pulse and species of the co-occurring native plants [30–32].

In the current study, the growth response of *A*. *retroflexus* and *G*. *max* to N pulses was different. In pure culture, *A*. *retroflexus* had a greater growth response to N pulses (the SP and DP treatments) than *G*. *max*. The total biomass of *A*. *retroflexus* was 1.5–40% greater (*P* < 0.05) in the SP and DP treatments compared to the NP treatment, whereas the total biomass of *G*. *max* was 0.8%-10% greater (*P* < 0.05) in the SP and DP treatments compared to the NP treatment. The NP treatment was unbeneficial while the SP and DP treatments were beneficial for *A*. *retroflexus* to accumulate biomass either in pure culture or mixed culture. The biomass of *A*. *retroflexus* was greatest in the SP treatment at the first harvest and greatest in the DP treatment at the last three harvests. Pure *G*. *max* had the greatest biomass in the DP treatment and lowest biomass in the NP treatment, while mixed culture had the greatest biomass in the NP treatment and lowest biomass in the SP treatment in mixed culture at most growth stages.

*A*. *retroflexus* and *G*. *max* had different seasonal dynamics of biomass production in response to N pulses which may be the result of different biological and life-history traits of the two species. *A*. *retroflexus* is a luxury consumer of N, increasing biomass with increasing N availability [33]. When N fertilizer is applied once on the planting date, *A*. *retroflexus* can efficiently uptake N in the early growth stages leading to rapid initial growth. Conversely, *G*. *max* is an abstemious consumer of N and growth may be adversely affected by the application of large amounts of N fertilizer [34]. However, there are two crucial growth stages of *G*. *max* related to N-fixation. One is the symbiosis development stage of *G*. *max*. At the early growth stage, N-fixation may be insufficient to meet the plants N requirements. The other is the pod filling stage during which nodule senescence may occur because the seed synthesis has a high photosynthetic demand [35]. Under these circumstances and when soil N is low, N-fertilizer could complement plant N demands [35]. Previous research [36] using the same experimental design found the maximum net photosynthetic rate (*Pmax*) of *A*. *retroflexus* is significantly higher than *G*. *max* in the seedling stage, whereas the *Pmax* of *G*. *max* was higher in the flowering and pod-filling stage across all three N treatments. The DP treatment coincides with the seasonal dynamics of *G*. *max* photosynthesis, providing needed N to meet the photosynthetic demand. As a result, *G*. *max* had the greatest biomass in pure culture in the DP treatment. For *A*. *retroflexus*, the SP treatment coincides with the seasonal dynamics of photosynthesis. As a result, *A*. *retroflexus* had the greatest biomass in the SP treatment in the earliest growth stage. In this experiment, mixed culture *G*. *max* produced the most biomass in the NP treatment, which could be because *G*. *max* is a legume, and leguminous seedlings may deal with intense competition from high N use efficiency C_4_ grasses (such as *A*. *retroflexus*) through N_2_ fixation [37]. When large amounts of N fertilizer are applied in early growth stages there is transient inhibition of nodule establishment [38]. Thus, mixed culture *G*. *max* produced the minimum biomass in the SP treatment (Fig 1).

### Responses to competition

Competition is one of the key drivers of species composition and community structure [39–40]. Competition includes all direct and indirect effects as well as facilitation and inhibition interactions which may change depending on resource availability and plant life history [41]. Compared with pure cultures, plant interactions in mixed cultures can have positive or negative effects on plant growth [27]. In the current study, we observed RYP_GA_ values above 1 at the last three harvests (*P* < 0.05), suggesting a positive responses of *G*. *max* to the existence of *A*. *retroflexus*. Our results disagree with previous research on the competitive effects of *A*. *retroflexus* on *G*. *max* yield [42–47], where *G*. *max* yield was unaffected or reduced by *A*. *retroflexus*.

Relative yield total (RYT) is an important parameter used in analyzing species interaction. RYT is the sum of the ratio changes in yield of the mixtures, and evaluates the extent to which the mixture of two-species requires the same resources [48]. In the current research, the RYT values of mixtures were mostly higher than one across the three N treatments (*P* < 0.05), suggesting partial resource complementarity occurred in the mixture of *A*. *retroflexus* and *G*. *max* (Fig 4). RYT values greater than one were also reported in other research of broadleaf weeds in *G*. *max* crops [49–51], due to different utilization of soil nutrient sources and different root and shoot characteristics. In the current research results have shown that N pulse, sampling date and the interactions have significant effects on RYT, indicating that the complementarity level is affected by N pulse (Table 2).

Light is an important resource effecting competitive ability of weeds and crops. Growth rate and height are both important traits crucial to competition for light [52]. *A*. *retroflexus* has smaller seedling but quickly out paces *G*. *max* because of its higher initial RGR. In later growth stages, *A*. *retroflexus* is generally taller than *G*. *max* and can shade it out [53].

Invasive plants can replace native plants due to higher competitive ability [53]. However, values of RYP_AG_ were lower than values for RYP_GA_ in this research (*P* < 0.05). Similar results have been found for other invasive weeds and native crops [54]. Invasive species do not always have a competitive advantage over native species [55]. The establishment and growth rates of invasive species are sometimes increased by accelerative or reciprocal interactions with native species [56]. Facilitation is considered to be one of the important factors of success for invasive species [57]. In the current study, we found *A*. *retroflexus* accelerated *G*. *max* growth across the three N treatments at the last three harvests, indicating that *A*. *retroflexus* has an adaptive capacity to reduce interspecific competition, which may accelerate its invasion to *G*. *max* crops in China.

## Conclusions

The results of this study support the first hypothesis that the invasive weed *A*. *retroflexus* had a superior growth response to N pulses (the SP and DP treatments) than *G*. *max* in pure culture. Greater biomass was recorded for *A*. *retroflexus* than *G*. *max* in the N pulse treatments. Our results also support the second hypothesis that when the two species are planted in mixture, *A*. *retroflexus* has superior competitive ability in N pulses conditions. In mixture, we found biomass of *A*. *retroflexus* was greater in N pulse conditions while biomass of *G*. *max* did not differ among the three N treatments at the first harvest. In the subsequent harvests the biomass of *G*. *max* was higher in the NP treatment compared to the SP and DP treatments. Overall, our results are in agreement with the FARH, which states that invasion is accelerated by high resource availability due to disturbance.

In addition, we found biomass of both species was higher in mixed culture than in pure culture at most growth stages. Relative yield total (RYT) values were all greater than 1.0 at the last three harvests regardless of N treatment, indicating partial resource complementarity occurrs when *A*. *retroflexus* is grown with *G*. *max*. These results suggest that *A*. *retroflexus* has an adaptive capacity to reduce interspecific competition, which may accelerate its invasion to *G*. *max* cropland in China.
